# Supplementary material for: Diabetes with COVID-19 was a significant risk factor for mortality, mechanical ventilation, and renal replacement therapies: A multicenter retrospective study in Japan
Source: PLoS One. 2025 Mar 19;20(3):e0319801. doi: 10.1371/journal.pone.0319801 (PMC11922262; doi:10.1371/journal.pone.0319801)
Supplement: S1 Table — Abbreviations: DPP-4 = dipeptidyl peptidase 4, SGLT2 = sodium glucose co-transporter 2, AGI=Alpha-glucosidase inhibitor. (DOCX) [file pone.0319801.s001.docx]

S1 Table. Content of hypoglycemic agents for patients with diabetes. Abbreviations: DPP-4=dipeptidyl peptidase 4, SGLT2=sodium glucose co-transporter 2, AGI=Alpha-glucosidase inhibitor

|  | Ratio (%) | total (N) |
| --- | --- | --- |
| DPP-4 inhibitors | 42.4 | 1573 |
| Biguanide | 19.9 | 739 |
| SGLT2 inhibitors | 15.4 | 573 |
| AGI | 7.3 | 272 |
| sulfonylurea | 7.0 | 258 |
| Glinides | 4.3 | 158 |
| thiazoline | 3.7 | 139 |
| total | 100.0 | 3712 |
