# Supplementary material for: Diabetes with COVID-19 was a significant risk factor for mortality, mechanical ventilation, and renal replacement therapies: A multicenter retrospective study in Japan
Source: PLoS One. 2025 Mar 19;20(3):e0319801. doi: 10.1371/journal.pone.0319801 (PMC11922262; doi:10.1371/journal.pone.0319801)
Supplement: S2 Table — Abbreviation: BMI=Body mass index. (DOCX) [file pone.0319801.s002.docx]

S2 Table. Multiple regression analysis of mortality adjusted with each factor, diabetes, age, sex (male), BMI, and ambulance. Abbreviation: BMI=Body mass index

|  | β | Odds ratio | 95% confidence interval | p value |
| --- | --- | --- | --- | --- |
| diabetes | 0.98 | 2.67 | 2.169-3.281 | <0.01 |
| ambulance | 0.86 | 2.36 | 1.938-2.870 | <0.01 |
| sex (male) | 0.57 | 1.77 | 1.426-2.185 | <0.01 |
| age | 0.95 | 1.10 | 1.089-1.110 | <0.01 |
| BMI | 0.06 | 1.06 | 1.034-1.085 | <0.01 |
